# Knowledge and skill: a case for ontological equality

**DOI:** 10.3389/fnhum.2013.00916

**Published:** 2013-12-26

**Authors:** Ludger van Dijk, Raoul M. Bongers

**Affiliations:** University of Groningen, University Medical Center Groningen, Center for Human Movement SciencesGroningen, Netherlands

**Keywords:** skill, knowledge, ontology, neuroscience, situated cognition, embodiment

In this commentary on “Motor skill depends on knowledge of facts” by Stanley and Krakauer (2013) (henceforth, S&K) we aim to sketch an apparent contradiction in S&K's argument on the dependence of skills on knowledge of facts. We contend that S&K's plea for this dependence stems from another form of independence of knowledge and skills—namely an “ontological” independence. We show what this means by introducing the difference between theories that are hierarchically organized, where one level has priority over another level, and theories that do not assume such an organization. We believe neuroscience and psychology have a lot to gain by taking note of these distinct attitudes, as they lead to radically different directions of inquiry and explanation. We shall show this by explicating the attitudes throughout S&K's argument, as that will go toward resolving the apparent contradiction.

In their thought provoking article S&K call into question the generally accepted view that skills are independent of knowledge of facts. After sketching a historical and philosophical context, S&K provide clarification of often confusingly applied notions as propositional or declarative knowledge, perceptual acuity, and the likes. The authors carefully argue against giving necessary and sufficient conditions for either skills or knowledge. To make their claim against independence S&K propose, to our delight, to look for knowledge and skill within the situation in which they are shown.

In making their claim, S&K seem to draw an analytical distinction between knowledge of facts and skills. As agents always need to “know what to do to initiate the actions that manifest a skill” (p. 5) (1), S&K assert that skills cannot be said to be independent of knowledge of facts. Neuroscientific data too, such as those from studying HM, are taken to show that HM always needed “explicit” instructions and needed to “use that knowledge each time” (p. 8) in order to learn a skill. In fact, because HM did not show his skills without these instructions, he cannot be properly said to have skill at all. Rather, he has what S&K call “motor acuity.”

With this reasoning S&K however, do not only argue against the independence of knowledge and skill, they make the stronger claim that knowledge is always *prior* to skill. They assert that knowledge is minimally a state with propositional content used for guiding actions (p. 1). Together, knowledge thus, works as a distinct state that first initiates and subsequently guides motor acuity. As such knowledge is treated as an isolated state that, together with motor acuity, underlies skill. So although S&K claim knowledge and skill should not be taken to be independent, their account shows that the dependence is a superficial one. That is, S&K argue for physical dependence by creating ontological independence. Not only are knowledge and skill thus, still independent entities, *ontologically* knowledge is even prioritized. It is this perspective that prompts S&K's conclusion that skills depend on knowledge of facts.

Although we are sympathetic to the claim that skills and knowledge of facts are strongly dependent notions, it is this ontological priority we aim to argue against here. We will start with a brief correction of S&K's historical overview, as we believe it both shows and propagates a misunderstanding at the heart of their view. Subsequently, we will argue for a point that S&K did not recognize, namely that the tradition of Merleau-Ponty, Wittgenstein, and Dreyfus was aimed at overcoming exactly the tendency to find ontological priorities. That is, for them knowledge and skills stand on equal footing, rather than the one underlying the other. Once we showed this, S&K's reading of these philosophers is easily identified as inappropriate. On a proper reading the question of (ontological) priority should not come up. Finally, we assert that taking Dreyfus seriously indeed makes a good argument against the independence of skills and knowledge of facts, and from this perspective S&K's re-interpretation of HM and other neuroscientific data offer a new look at neuroscientific literature. However, we shall argue that this is not, as S&K suggest, because we finally free neuroscience from the influence of the “predominant” 20th century tradition. Rather, it is by finally embracing such a tradition that neuroscientific data can be seen afresh.

## Modern philosophy and neuroscience

Before moving on to our main argument, it is worth pausing at one of the historical claims S&K made. They assert that the anti-cognitivist view of Dreyfus, which follows the tradition of Merleau-Ponty and Bourdieu (and we may add Heidegger and Wittgenstein) is in fact the dominant view in philosophy and the social sciences, and that neuroscience mirrors this philosophical literature (p. 2). Their overview suggests thus, that neuroscientific theorizing is held hostage by an anti-cognitivist perspective that separates knowledge of facts from skill and empirical studies in neuroscience do no more than mirror this philosophical thesis.

This, we believe, is a false rendition of the history of cognitive neuroscience and its psychological and philosophical antecedents. The dissociation of knowledge of facts from skill in neuroscientific literature echoes the distinction between (perception), cognition and action that comes with the dominant computer metaphor (of input, processing, and output) of the 1960's onward (see e.g., Posner and DiGirolamo, 2000; Hurley, 2002; Boden, 2006). The computationalist view that cognition is the computational manipulation of representations (e.g., Newell and Simon, 1976) in turn has its roots in Cartesian philosophy of the 17th century that placed the mind in a the mechanistic body (Boden, 2006). This idea was propagated in philosophy, and re-affirmed in psychology as thought and action were made to fit the emerging psychophysical methods (through e.g., Wundt and Titchener), ending up with cognition as an invisible internal state that constructs percepts from incoming sensations, and coordinates movements by outgoing motor commands.

Notice how in this historical picture theorizing is informed by a belief that for understanding the mind, it makes sense to look for underlying elements that cause it. Direction of inquiry is thus, vertically directed. For example, perception is made up of underlying elementary sensations, and skills are nothing but movements guided by cognitive commands (what S&K would call “knowledge of facts”). It is exactly this analytical, intellectualist attitude that Dreyfus, Merleau-Ponty, but also Ryle and Wittgenstein, each in their own way, aimed to displace. But their role has thus, certainly never made the impact on (cognitive) neuroscience S&K claimed it does. To date it has been limited to but a view prevailing non-representational or non-computational approaches to psychology (e.g., Gibson, 1979; Thelen et al., 1994; Kelso, 1995; Reed, 1996; Chemero, 2009).

## A horizontal approach

So much for the groundwork, now on to our main argument, because the view S&K claim Dreyfus' tradition holds is itself also misguided. For this we find it useful to distinguish two basic ways of directing inquiry in philosophy (and psychology). First, there is the attitude that we just exemplified in the preceding section. It roughly conceives the world to be composed of supervening layers, e.g., going up from atoms to cells to brains and minds. This is often associated with reductionism (though it need not be), internalism about mental life, and with physicalism; conceiving of cognitive states—like knowledge—as something you have as a (physical, informational) state or process. Elsewhere, we have called this approach to psychology a “vertical worldview,” as it shows a tendency to explain (empirical) phenomena by analyzing downward to underlying (and often hidden and abstract) essentials (van Dijk and Withagen, 2014).

In contrast to a vertical worldview, Wittgensteinian and Heideggerian traditions approach their subject more horizontally. Metaphorically, this attitude does not start out with a layered structure, and phenomena are not relocated along a vertical axis, but keeps to a horizontal plane. That is, the attitude resists the urge to analyze beyond the phenomena in search for essence, and locates both large and small scale phenomena at the same level (van Dijk and Withagen, 2014). This means that understanding phenomenon requires seeing in what particular, concrete situations it actually does or does not play a role. To explain a phenomenon, such as knowledge, a horizontal approach thus, looks at the particular, concrete situation in which it actually comes up, rather than treating all particular cases as similar and trying to derive abstract underlying essences from that (see also Wittgenstein, 1969, § 10).

Importantly, a horizontal approach does not deny the reality of cognitive states or any other aspect of human life typically assigned to lower levels of description. However, it does deny this re-conceptualizing of knowledge as a state below apparent behavior. So, for example, Merleau-Ponty's denial that skilled behavior manifests cognitive states (p. 2) is not a denial of experts having knowledge, but a denial of the identification of cognition (knowledge) with an underlying (guiding) state. In short, much like S&K, the horizontal approach aims to direct attention to the concrete performances of skill in particular situations to explain knowledge of facts. However, the focus of inquiry remains with these concrete performances and does not subsequently analyze to an ontological priority beneath it.

It is much more fruitful, we feel, to also read Dreyfus' work from this horizontal perspective. In his phenomenological analysis of skill acquisition, Dreyfus brings to view the fact that as one learns, one grows into a concrete situation; getting more in touch with the world, rather than abstracting away from it by constructing abstract rules (e.g., propositional knowledge) to guide engagement. To Dreyfus, skill acquisition is not a vertically directed process of going from concrete sensorimotor couplings (e.g., Piaget, 1954) upwards and inwards to abstract generally applicable rules. Rather, skill acquisition moves horizontally from abstract instructions (because they lack application) to concrete, highly adaptable, perceptual-motor behavior.

Thus, from a horizontal approach Dreyfus' assertion that expertise does not require unconscious rules should not be read as a plea against experts having knowledge, but against assigning knowledge one level below concrete behavior to a hidden state (with unconscious propositional content). That experts do not fall back on explicit rules when performing therefore, does not mean that they lack knowledge or are not knowledgeable, in fact, it shows that they have knowledge galore. Interestingly, S&K argue basically the same, however, they feel the urge to subsequently suppose that having this expert knowledge requires a hidden layer of propositional content. Dreyfus' horizontal attitude, by contrast, resists such an analytical abstraction away from the actual phenomenon.

We believe that because S&K have given an overly vertical reading of Dreyfus, their argument misses the mark. Their rendition of the historical and ontological commitment of Dreyfus' tradition shows that the authors might themselves be deeply influenced by an intellectualist, vertical approach to psychology and (cognitive) neuroscience. Because of this S&K failed to see how close to Dreyfus they actually get.

## Concluding remarks

In this short commentary we hope to have shown the limits of S&K's analysis of the relation between skills and knowledge and its history in philosophy. We did so by pointing to an ontological distinction between vertical and horizontal approaches to the subject. We believe a study of skills, knowledge, and any other aspect of human behavior has much to gain from considering a horizontal approach. The horizontal view on skill acquisition and the role of perceptual and motor acuity has for example important consequences for developing theories and hypotheses in motor control and important implications for neuroscientific research.

We believe that S&K have offered us a compelling empirical argument against the independence of skills and knowledge and an important re-interpretation of seminal neuroscientific literature. They showed that both knowledge and skills are aspects of one and the same world of everyday life. But rather than dismissing Dreyfus' tradition, we hope to have shown that they ought to embrace the tradition fully to make their claim against independence. Maybe this will inspire neuroscience to consider a horizontal approach to their role in psychology. This, we feel, would have mutually beneficial effects for both psychology and neuroscience.

## Notes

1. All page numbers refer to Stanley and Krakauer (2013).
